# One-pot synthesis of Au/GO nanozymes: enhanced catalytic activity *via in situ* chemical reduction

**DOI:** 10.1039/d6ra00491a

**Published:** 2026-03-17

**Authors:** Mohamed A. Abdelgawad, Jumana A. Sanari, Mohammed Gamal, Ola G. Hussein

**Affiliations:** a Department of pharmaceutical chemistry, College of pharmacy, Jouf University Sakaka 72388 Saudi arabia; b Joseph Black Building, School of Chemistry King's Buildings, David Brewster Road Edinburgh EH9 3FJ UK; c Department of Pharmaceutical Analytical Chemistry, Faculty of Pharmacy, Beni-Suef University Alshaheed Shehata Ahmed Hegazy St. 62574 Beni-Suef Egypt; d Department of Pharmaceutical Chemistry, Faculty of Pharmacy, Future University in Egypt Cairo 11835 Egypt ola.farag@fue.edu.eg

## Abstract

Synthetic nanozyme materials are engineered to mimic the catalytic activities of naturally occurring enzymes making them highly appealing because they are more stable, cheaper to produce, and offer easily customizable catalytic characteristics compared with the enzyme systems found in nature. In this work, we present the fabrication of a gold nanoparticle-decorated graphene oxide (Au/GO) nanocomposite using an *in situ* chemical reduction method. Graphene oxide served as a high-surface-area scaffold enabling the uniform anchoring of gold nanoparticles while sodium borohydride facilitated efficient reduction of HAuCl_4_ to Au^0^. Comprehensive characterization using SEM, TEM, EDX, and FT-IR and UV-vis spectroscopies verified the successful synthesis of the well-dispersed heteroatom-enriched nanocomposite with well-defined porosity and favorable optical attributes. Au/GO-NPs demonstrated significant peroxidase-like catalytic behavior by efficiently promoting the colorimetric oxidation of *o*-phenylenediamine (OPD) in the presence of hydrogen peroxide achieving a wide linear detection range (5–700 µM) and a low detection limit of 4.03 µM. Coupled with the glucose oxidase (GOx) system, Au/GO-NPs effectively detected glucose through the *in situ* generation of hydrogen peroxide providing consistent measurements within the range of 50–700 µM. Kinetic evaluation yielded a Michaelis constant (*K*_m_) of 0.589 mM and a maximum reaction rate (*V*_max_) of 1.19 × 10^−3^ µM/min reflecting efficient enzyme-mimicking activity of the system. The Au/GO-NP-based colorimetric glucose sensor displayed excellent selectivity against common interfering substances and outstanding reproducibility (RSD <1.5%). These findings highlight the potential of Au/GO-NPs as a cost-effective and highly sensitive nanozyme platform for colorimetric biosensing particularly for the detection of hydrogen peroxide and glucose.

## Abstract 2

A cascade catalytic system combining glucose oxidase and an Au/GO nanozyme enables colorimetric glucose detection *via* enzymatic generation of hydrogen peroxide followed by nanozyme-catalyzed oxidation of *o*-phenylenediamine.

## Graphical abstract

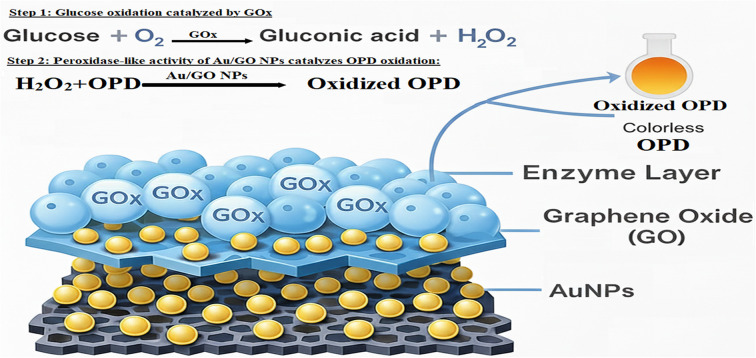

## 1 Introduction

Nanotechnology continues to play a transformative role in catalysis, biosensing, and environmental monitoring due to the remarkable physicochemical properties of nanomaterials particularly their high surface-to-volume ratio, tunable optical properties, and advanced catalytic efficiency.^1–3^ In this area of research, nanoscale materials crafted to perform enzyme-like catalytic functions have become highly valued because they exhibit remarkable stability and can operate in diverse conditions and allow precise adjustment of their functional behavior.^4–7^ Unlike traditional enzymes, nanozymes display enhanced resistance to extreme pH, elevated temperatures, and enzymatic degradation making them well-suited for practical applications in medical diagnostics, environmental sensing, and industrial catalysis.^8–15^

Recently, pharmaceutical compounds have been explored as novel precursors for constructing functional nanomaterials due to their inherent heteroatom-rich structures. Graphene oxide (GO), a two-dimensional carbon-based material enriched with oxygen-containing functional groups and provides an excellent substrate for the construction of multifunctional nanocomposites.^16–18^ Its abundant hydroxyl, epoxy, and carboxyl groups facilitate metal-ion adsorption acting as nucleation sites for nanoparticle growth.^19^ GO also exhibits high surface area and favorable electronic properties making it an ideal platform for hybrid nanozyme engineering.^20–24^

Gold nanoparticles (AuNPs) are well-known for their high biocompatibility, excellent catalytic activity, efficient electron-transfer characteristics, and strong affinity toward biological molecules.^25–28^ Incorporating AuNPs onto GO sheets leads to the formation of Au/GO hybrid nanozymes exhibiting a synergistic catalytic behavior that is superior to that of either the components alone.^29^ The enhanced peroxidase-like performance of Au/GO systems stems from improved electron transport, increased reactive surface sites, and strengthened substrate interactions.^30,31^

Au/GO-NPs demonstrated pronounced peroxidase-mimicking catalytic behavior effectively catalyzing the colorimetric oxidation of *o*-phenylenediamine (OPD) in the presence of hydrogen peroxide (H_2_O_2_). This catalytic response enabled sensitive and selective detection of hydrogen peroxide across a broad concentration range with a low detection limit of 4.03 µM. Furthermore, the system was coupled with glucose oxidase (GOx) nanozymes and was employed for glucose sensing *via in situ* hydrogen peroxide generation. The hybrid biosensing platform showed consistent performance in detecting glucose concentrations in the range of 50–700 µM alongside excellent reproducibility (RSD <1.5%) and resistance to common interfering agents.

These findings demonstrate that Au/GO-NPs hold strong promise as an effective and economical nanozyme platform with practical applications in biosensing. The integration of heteroatom doping, metal activation, and template induced porosity offers a strategic approach to designing multifunctional nanozymes suitable for sensitive analytical platforms especially for glucose and hydrogen peroxide detection in biomedical diagnostics.

## 2. Experimental

### 2.1. Analytical devices and measurement tools

The fabricated Au/GO-NPs were characterized for surface features using imaging with a Quanta FEG-250 scanning electron microscope (SEM) and Transmission Electron Microscope (TEM), JEM 2100, Japan). Elemental analysis and confirmation of heteroatom and metal incorporation within the nanoparticles were carried out *via* energy-dispersive X-ray spectroscopy (EDX). UV-vis absorption measurements were carried out using a UV-1900i PC instrument from Kyoto, Japan operated through the UV Probe software (v2.43). Spectral data were acquired using 1 cm quartz cuvettes with measurements taken at a scanning speed of 2800 nm/min and a bandwidth of 1 nm. To identify functional groups and investigate chemical bonding within the nanostructure, Fourier transform infrared (FT-IR) spectra were collected using a Shimadzu IR 435 spectrometer (Shimadzu Corp., Kyoto, Japan).

### 2.2. Reagents and materials used

Ultrapure-grade water supplied by an ELGA PURELAB Flex purification apparatus (Model PF3XXXXM1, UK) and used throughout for all experiments. Graphene oxide (GO), chloroauric acid (HAuCl_4_·3H_2_O), and sodium borohydride (NaBH_4_) were acquired from Sigma-Aldrich (Germany). These reagents were used without any further purification. A set of experiments was conducted in an acetate-buffered medium at pH 4.0 to evaluate the enzyme-like peroxidase activity of the synthesized nanoparticle system. Hydrogen peroxide (H_2_O_2_) and glucose oxidase also sourced from Sigma-Aldrich were employed in catalytic performance tests. For biological experiments, human serum samples were acquired under formal permission from VACSERA the Holding Company for Biological Products and Vaccines in Giza, Egypt.

### 2.3. Methodology for synthesizing Au/GO Nanoparticles

Graphene oxide (20 mg) was dispersed in 40 mL of deionized water and sonicated for 1 h to obtain a stable suspension. HAuCl_4_ solution (0.5–1.0 mM) was added dropwise under stirring to allow Au^3+^ adsorption onto the GO functional groups. A fresh ice-cold NaBH_4_ solution (10-fold excess) was subsequently introduced to reduce Au^3+^ to metallic Au^0^. The mixture changed from brown to dark red confirming the formation of Au NPs. After 1 h of reaction, the product was centrifuged and washed repeatedly to remove residual ions yielding purified Au/GO nanozymes.

### 2.4. Characterization of Au/GO nanoparticles (Au/GO-NPs)

A variety of analytical techniques were employed to investigate the structural and chemical features of the synthesized Au/GO-NPs and to evaluate their performance for hydrogen peroxide detection through a colorimetric approach based on peroxidase-like activity alongside an analysis of their surface structure and particle dispersion using SEM and TEM. Energy-Dispersive X-ray Spectroscopy (EDX) was utilized to determine the elemental composition and confirm the uniform distribution of C, O, and Au throughout the nanoparticles. Fourier Transform Infrared (FTIR) spectroscopy was conducted to identify the functional groups present and verify the successful doping of heteroatoms into the carbon framework. Furthermore, UV-visible spectroscopy was employed to examine both the light absorption behavior and catalytic activity of Au/GO-NPs particularly by monitoring the catalytic oxidation of OPD in the presence of hydrogen peroxide. Overall, the evaluation techniques established the successful formation of well-defined structures and catalytically active Au/GO-NPs highlighting their potential as robust nanozymes for biological detection and analytical measurements.

### 2.5. Au/GO-NP-based peroxidase mimetic approach for hydrogen peroxide detection

The catalytic activity of the synthesized Au/GO nanoparticles to mimic peroxidase was examined by observing the oxidation of OPD in the presence of hydrogen peroxide using UV-visible spectroscopy. A quartz cuvette was loaded with 185 µL of acetate buffer (0.2 M, pH 4.0) to which 10 µL of Au/GO nanoparticle stock (1 mg/mL), 24 µL of H_2_O_2_ (100 mM), and 24 µL of OPD solution (60 mM) were subsequently added to prepare the reaction mixture. The mixture was incubated at 40 °C for 10 minutes. UV-vis absorbance was then measured from 300.0 to 600.0 nm at 450.0 nm serving as an indicator of OPD oxidation. A parallel control experiment performed in the absence of hydrogen peroxide confirmed that the catalytic reaction was exclusively mediated by Au/GO nanoparticles.

### 2.6. pH-dependent catalytic activity of Au/GO-NPs

The catalytic efficiency of Au/GO nanoparticles under different pH conditions was evaluated using 0.2 M acetate buffers ranging from pH 2.0 to 8.0. The reaction involved monitoring the oxidation of OPD with the absorbance of the resulting product at 450.0 nm used to determine how varying pH levels influence the peroxidase-like activity of nanozymes.

### 2.7. Evaluating the role of temperature in Au/GO-NPs catalysis

To evaluate the effect of temperature on Au/GO nanoparticle catalysis, reactions were performed in tightly controlled water baths at temperatures ranging from 20 °C to 80 °C. The absorbance of the oxidized product at 450.0 nm was used to quantify their peroxidase-like activity while all other experimental parameters remained unchanged.

### 2.8. Time-dependent catalytic behavior of Au/GO nanoparticles

To explore how the incubation time influences the catalytic efficiency of Au/GO nanoparticles, the reactions were conducted for incubation times ranging from 2 to 20 minutes. The goal was to determine the incubation time that delivers the most consistent and strong response facilitating glucose detection with higher precision and dependability.

### 2.9. Calibration of Au/GO-NPs for hydrogen peroxide sensing

A calibration analysis was conducted to examine how effectively the Au/GO nanoparticle-based sensor could detect and quantify the target substance. Initially, the 1 mg/mL Au/GO NPs stock solution was scanned over the wavelength range of 300.0–600.0 nm to determine the maximum absorption wavelength (*λ*_max_) under standard conditions. Next, hydrogen peroxide solutions at different concentrations were prepared using the previously described protocol. The amount of oxidized substrate in each solution was determined from its 450.0 nm absorbance and the resulting data were plotted to generate the calibration curve.

### 2.10. Kinetic study of the catalytic performance of Au/GO-NPs

A kinetic study was performed to assess the peroxidase-like activity of Au/GO-NPs using OPD as the substrate. The nanoparticle concentration was fixed at 1 mg/mL while OPD concentrations ranged from 0.1 to 50 mM. The initial reaction rates were determined from the increase in absorbance at 450.0 nm and analyzed using the Michaelis–Menten model to obtain *K*_m_ and *V*_max_. For context, the kinetic performance of Au/GO-NPs was compared with natural horseradish peroxidase (HRP)^32^ and previously reported nanozyme systems such as FeCu@CDs^33^ and Au-Ag-Pt NPs.^34^ This comparison provides a framework to evaluate the substrate affinity and catalytic behavior of Au/GO-NPs relative to established enzyme and nanozyme systems.

### 2.11. GOx-Au/GO-NPs coupled system for glucose detection

Glucose detection was achieved through a sequential enzymatic and nanozyme approach. First, varying concentrations of glucose were incubated with 20 µL of glucose oxidase (40 IU per mL) in 50 mM acetate buffer at pH 5.1 for 30 minutes at 37 °C allowing glucose to be enzymatically converted into hydrogen peroxide. Next, the mixture was supplemented with 24 µL OPD (60 mM), 10 µL Au/GO nanoparticles (1 mg/mL), and 800 µL acetate buffer (0.2 M, pH 4.0). The H_2_O_2_ formed in the first step induced OPD oxidation through the peroxidase-like activity of nanoparticles, resulting in a visible color change proportional to the glucose level.

### 2.12. Selectivity assessment of the Au/GO nanoparticle-based glucose sensor

Glucose selectivity of Au/GO-NPs sensor was examined *via* testing its response in the presence of several common biological molecules. Citric acid, sucrose, lactose, uric acid, ascorbic acid, and fructose were introduced at physiologically relevant concentrations to evaluate their impact on the assay signal. The results demonstrated that Au/GO-NPs system specifically recognizes glucose showing minimal interference from these other substances.

### 2.13. Application to real samples for glucose measurement

The practical utility of Au/GO-NPs colorimetric sensor was demonstrated by analyzing glucose levels in human serum samples obtained from commercial sources. Serum samples were first brought within the measurable range of the sensor by diluting them with 0.2 M acetate buffer at pH 4.0. Glucose levels were then quantified using the GOx-Au/GO-NPs cascade method. Comparison with a conventional glucometer confirmed that the sensor provided highly consistent results. To further validate accuracy, recovery studies were performed by spiking serum samples with known glucose concentrations yielding reliable and consistent results that confirm the effectiveness of the method for real-world applications.

## 3 Results and discussion

### 3.1. Structural and compositional examination of Au/GO-NPs

A comprehensive characterization of the synthesized Au/GO nanoparticles was performed to investigate their morphology, composition, and structural properties. Techniques employed for this purpose included SEM, TEM, EDX, UV-visible spectroscopy, and FTIR spectroscopy.

**Fig. 1:**
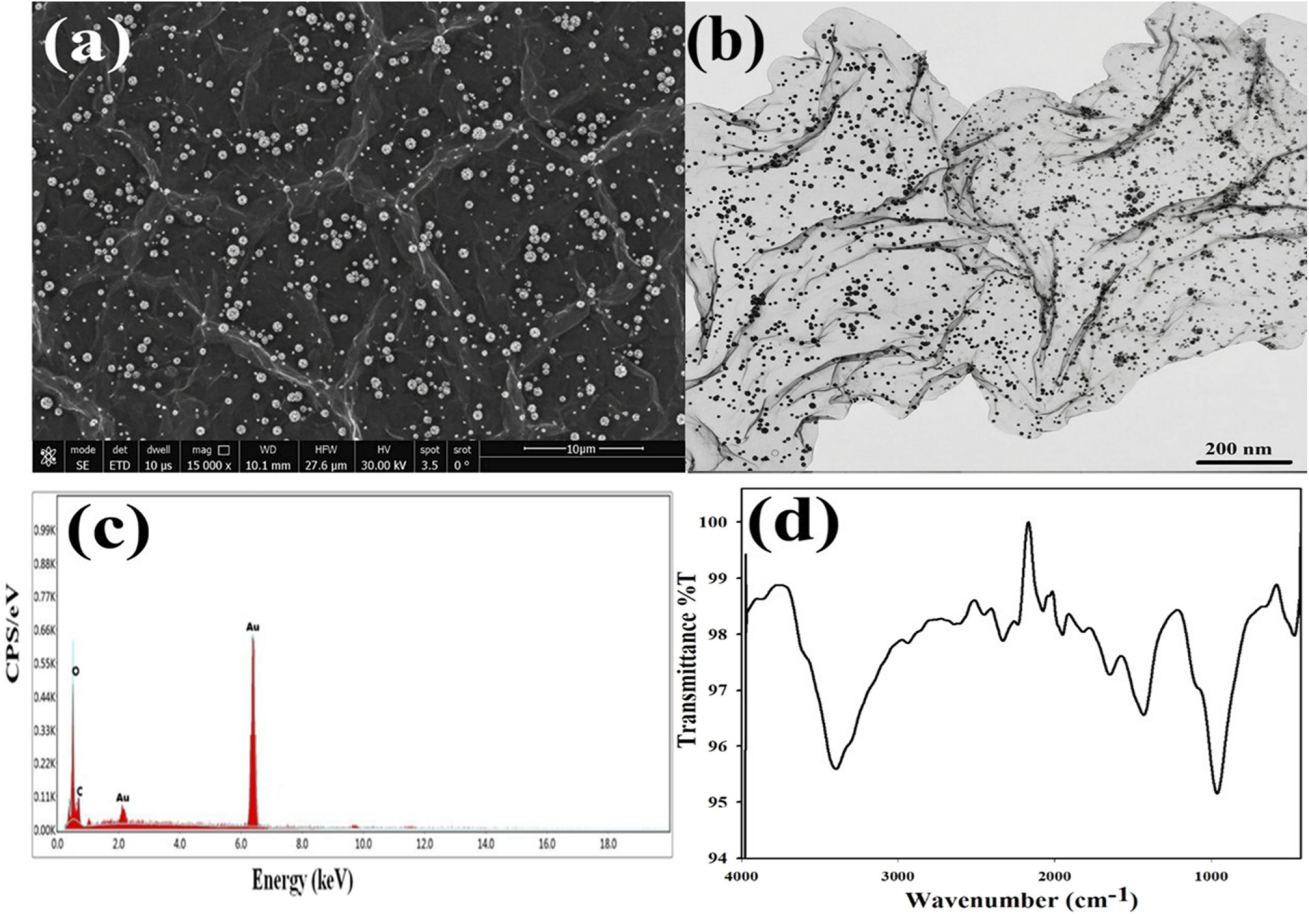
(a) SEM image of Au/GO showing the Au nanoparticles uniformly distributed on the crumpled graphene oxide sheets. (b) TEM image illustrating the sheet-like and wrinkled morphology of graphene oxide in the Au/GO nanocomposite. (c) EDX spectrum indicating the presence of Au, along with carbon and oxygen in the nanocomposite. (d) FT-IR spectrum of Au/GO showing characteristic GO functional groups and their interactions with Au nanoparticles.

The successful formation and morphology of the gold/graphene oxide (Au/GO) nanocomposite were confirmed through electron microscopy analysis. The SEM image, as shown in Fig. 1a, provided a topographical overview of the composite revealing the characteristically wrinkled and crumpled sheet-like structure of the graphene oxide (GO) substrate. The bright high-contrast spots observed across the entire surface correspond to gold nanoparticles (Au NPs) indicating excellent coverage and anchoring of the metallic phase onto the insulating GO support. This low-magnification view confirms that the synthesis method successfully decorated the large two-dimensional carbon matrix with gold features maintaining the crucial high surface area of GO.

Further structural confirmation was achieved *via* transmission electron microscopy (TEM) as shown in Fig. 1b. The TEM image reveals semi-transparent graphene oxide (GO) sheets decorated with numerous dark spherical nanoparticles corresponding to Au nanoparticles (Au NPs) which appear darker due to the higher electron density of gold. The Au NPs are well distributed across the GO surface with minimal aggregation indicating effective anchoring of nanoparticles on the GO sheets. This uniform dispersion suggests that GO provides abundant nucleation sites for Au NP formation. Such morphology is beneficial for increasing the available surface area and is advantageous for catalytic and biomedical applications. EDX analysis confirmed the elemental composition of the composite. The spectrum shown in Fig. 1c clearly exhibited three primary signals: a strong peak for carbon (C), a peak for oxygen (O), and distinct characteristic signals for gold (Au). The co-detection of C, O, and Au validates the successful formation of the Au/GO nanocomposite. Furthermore, the semi-quantitative analysis derived from the peak intensity ratios provides a direct assessment of the gold loading efficiency onto the graphene oxide support affirming the intended composition and purity of the material. The FTIR results (Fig. 1d) corroborated the formation of Au/GO-NPs through the appearance of signature vibrational bands associated with nitrogen- and sulfur-containing functional groups.

**Fig. 2:**
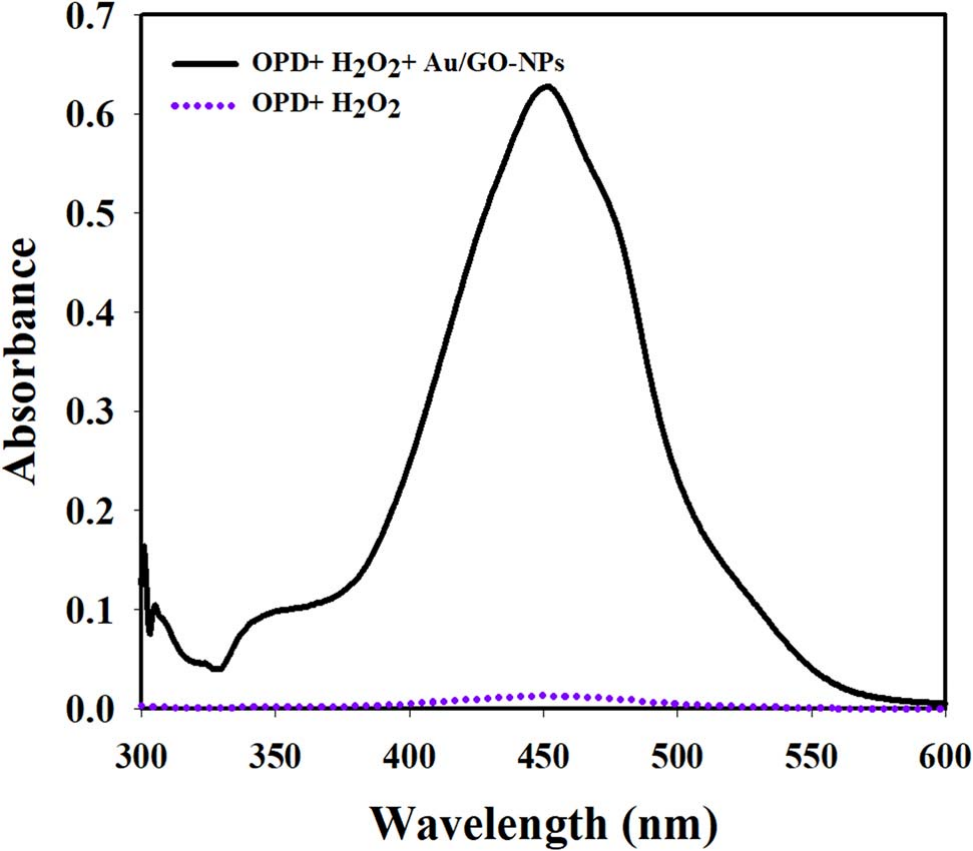
UV-vis absorbance spectra showing the catalytic effect of Au/GO nanoparticles (Au/GO-NPs) on the oxidation of OPD by hydrogen peroxide. Black line represents OPD + H_2_O_2_ + Au/GO-NPs, showing a pronounced peak at 450 nm while the purple dashed line (OPD + H_2_O_2_) shows negligible absorbance indicating minimal reaction in the absence of nanoparticles.

Finally, UV-vis spectra (Fig. 2) revealed specific absorption bands primarily attributed to π–π* and *n*–π* electronic transitions associated with conjugated nitrogen and sulfur moieties further validating the successful synthesis of Au/GO nanostructures.

### 3.2. Enzyme-mimicking catalytic activity of Au/GO nanoparticles

**Scheme 1:**
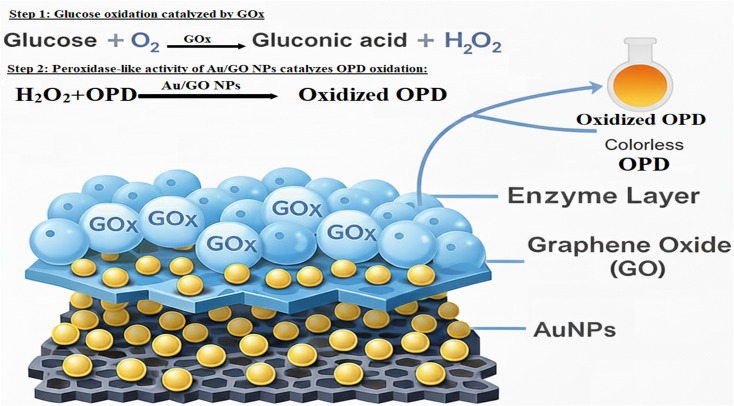
Schematic of the colorimetric sensing mechanism based on Au/GO nanoparticles (Au/GO-NPs). Glucose is first oxidized by glucose oxidase (GOx) in the presence of oxygen to produce gluconic acid and hydrogen peroxide. Subsequently, Au/GO-NPs exhibit peroxidase-like activity and catalyze the oxidation of *o*-phenylenediamine (OPD) by the generated hydrogen peroxide forming the colored oxidation product (DAP). Au/GO-NPs consisting of gold nanoparticles uniformly anchored on a graphene oxide scaffold provide abundant active sites and enhanced catalytic efficiency enabling the sensitive and selective colorimetric detection of hydrogen peroxide and glucose.

The catalytic function of Au/GO nanoparticles resembling that of peroxidase enzymes was investigated by analyzing their ability to drive the oxidation of OPD in the presence of H_2_O_2_. Here, hydrogen peroxide served as the main reactant while OPD provided a colorimetric signal upon oxidation. The reaction proceeds *via* a ping-pong mechanism analogous to the activity of horseradish peroxidase. Gold centers within Au/GO-NPs facilitate the decomposition of H_2_O_2_ into reactive oxygen species which then convert OPD into 2,3-diaminophenothiazine (DAP) producing a distinct yellow coloration. This oxidation process was monitored spectrophotometrically showing a time-dependent increase in absorbance at 450.0 nm indicative of product formation and catalytic efficiency. The color change to yellow-orange further confirmed the catalytic conversion of OPD. The reaction mechanism is likely driven by a Fenton-like pathway in which the electronic properties of gold nanoparticles and the high surface area of the graphene oxide support collectively promote electron transfer enhancing H_2_O_2_ decomposition and facilitating OPD oxidation. These results demonstrate the powerful peroxidase-mimicking performance of the Au/GO-NP system; Scheme 1.

### 3.3. Fine-tuning the catalytic performance of Au/GO nanoparticles

#### 3.3.1 pH-dependent catalytic performance of Au/GO nanoparticles

**Fig. 3:**
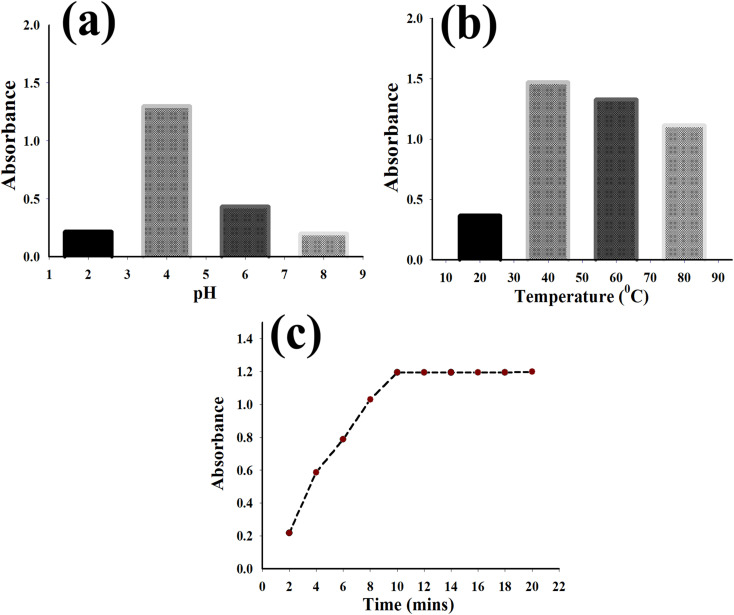
Effect of experimental parameters on the absorbance response of Au/GO nanoparticles (Au/GO-NPs). (a) Influence of pH on the absorbance of Au/GO-NPs, showing the maximum response at pH 4.0. (b) Effect of temperature on the absorbance of Au/GO-NPs, showing enhanced signal intensity at 40 °C. (c) Time-dependent absorbance profile of Au/GO-NPs, demonstrating a rapid increase followed by a plateau confirming the attainment of the reaction equilibrium at 10 min.

Catalysis by Au/GO nanoparticles was strongly influenced by the pH of the solution. Optimal peroxidase-mimicking behavior occurred under mildly acidic conditions (pH 4) suggesting this environment best facilitates the interaction of hydrogen peroxide and OPD with the nanoparticle surface (Fig. 3a).

Under these acidic conditions, the graphene oxide (GO) surface may be optimally protonated potentially altering the surface charge and enhancing the electrostatic attraction and adsorption of hydrogen peroxide and OPD substrates near the Au active sites. However, below pH 4 catalytic activity declined likely due to excessive protonation which could lead to the desorption of substrates and proton-induced changes in the electronic structure of Au nanoparticles that deactivate the active sites. Conversely, alkaline environments generated due to the deprotonation of GO functional groups and the structural instability of the gold nanoparticles negatively impacted their function. Thus, maintaining a slightly acidic pH is critical for achieving optimal catalytic output.

#### 3.3.2 Evaluating the role of temperature in Au/GO-NPs catalysis

The catalytic performance of Au/GO nanoparticles was strongly influenced by temperature. Absorbance measurements showed a progressive increase with higher temperatures with peak activity occurring at 40 °C (Fig. 3b) indicating that this temperature provides optimal conditions for accurate and responsive detection.

#### 3.3.3 Time-dependent catalytic behavior of Au/GO nanoparticles

Investigation of reaction progression as a function of time revealed a gradual rise in absorbance from 2 to 20 minutes after which the signal plateaued (Fig. 3c). Based on this value, a 10 minutes incubation period was chosen as the optimal balance between reaction completion and time efficiency enabling reliable catalysis of OPD oxidation by Au/GO-NPs.

### 3.4. Establishing a quantitative method for hydrogen peroxide detection

**Fig. 4:**
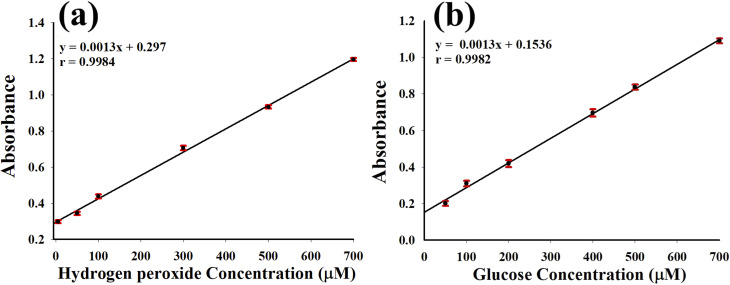
Calibration plots based on the absorbance response of Au/GO nanoparticles (Au/GO-NPs). (a) Linear relationship between absorbance and hydrogen peroxide concentration showing excellent linearity over the investigated range (5–700 µM). (b) Linear calibration curve for glucose determination using Au/GO-NPs with a high correlation coefficient demonstrating the suitability of the Au/GO-NP-based system for sensitive and reliable glucose sensing.

The Au/GO nanoparticles were assessed for their peroxidase-like activity to detect hydrogen peroxide, whose reactivity varies with concentration. Hydrogen peroxide plays a crucial role as a biomarker in clinical settings indicating oxidative stress linked to conditions such as cardiovascular diseases, neurodegenerative disorders, and cancer while also being important for environmental monitoring. Effective detection of hydrogen peroxide is crucial for medical diagnostics, therapeutic monitoring, and environmental evaluation. Using a 0.2 M acetate buffer (pH 4.0) and maintaining the system at 40 °C, the Au/GO nanoparticle-based sensor allowed efficient colorimetric measurement of hydrogen peroxide. A calibration curve generated from various concentrations (Fig. 4a) exhibited a linear range of 5–700 µM with a detection limit of 4.03 µM highlighting the potential of nanoparticles as an inexpensive and highly sensitive detection platform for both biomedical and environmental applications.

### 3.5. Color-based detection of hydrogen peroxide using Au/GO-NPs

Au/GO nanoparticles showed excellent enzyme-like catalytic performance enabling accurate measurement of hydrogen peroxide when tested under optimized conditions of 40 °C, pH 4, and a 10 min response time. The detection system displayed a strong linear correlation within the 5–700 µM range (Fig. 4a) and attained a detection threshold as low as 4.03 µM. This sensitivity confirms the practical potential of Au/GO-NPs as a reliable and economical sensor for hydrogen peroxide monitoring in clinical diagnostics and environmental assessments (Table 1).

**Table 1:** Parameters and analytical performance of the prepared Au/GO nanoparticle (Au/GO-NP)-based colorimetric sensor for hydrogen peroxide detection

Parameter	Value
Range (µM)	5–700

**Linearity**	
Slope	0.0013
Intercept	0.2970
Correlation coefficient (r)	0.9984
Incubation temperature	40 °C
Optimal pH	4
Optimal reaction time	10 minutes

### 3.6. Kinetic study of the catalytic performance of Au/GO-NPs

The peroxidase-like activity of Au/GO nanoparticles was evaluated through kinetic analysis using OPD as the substrate. A gradual increase in absorbance at 450.0 nm confirmed the catalytic oxidation of OPD and the reaction rate increased with substrate concentration indicating typical enzyme-like behavior. The initial reaction rates obtained from the linear portions of absorbance-time plots followed Michaelis–Menten kinetics. Fitting the data to the Michaelis–Menten model provided a *K*_m_ value of 0.5897 mM and a *V*_max_ of 1.19 × 10^−3^ µM/min. The relatively low *K*_m_ suggests good substrate affinity, while the observed *V*_max_ reflects the efficient catalytic activity of the Au/GO nanozyme supporting its potential as a peroxidase mimic. In comparison, natural horseradish peroxidase (HRP)^32^ exhibits a *K*_m_ of 0.051 mM and a *V*_max_ of 54.6 µM/min, while FeCu@CDs nanozymes^33^ have a *K*_m_ of 0.649 mM and a *V*_max_ of 8.5 × 10^−3^ µM/min, and Au-Ag-Pt NPs^34^ exhibited a *K*_m_ of 3.61 mM and a *V*_max_ of 14.68 × 10^−8^ µM/min for TMB oxidation; Table 2. Au/GO-NPs studied here show a *K*_m_ of 0.589 mM and a *V*_max_ of 1.19 × 10^−3^ µM/s. These comparisons allow for evaluation of the substrate affinity and catalytic performance of Au/GO-NPs relative to both natural enzymes and other nanozyme systems.

**Table 2:** Comparison of Au/GO nanoparticles with previously reported nanozymes for glucose detection

Nanozyme system	Substrate	*K*_m_ (mM)	*V*_max_	Reference
Horseradish peroxidase	OPD	0.051	54.6 µM/min	32
FeCu@CDs nanozyme	OPD	0.649	8.5 × 10^−3^ µM/min	33
Au-Ag-Pt NPs	TMP	3.61	14.68 × 10^−8^ µM/s	34
Au/GO-NPs	OPD	0.589	1.19 × 10^−3^ µM/min	This work

### 3.7. GOx-coupled Au/GO-NPs system for quantitative glucose analysis

Monitoring glucose with high accuracy is vital for diabetes and metabolic research. A stepwise colorimetric assay was developed by integrating glucose oxidase (GOx) with Au/GO nanoparticles. Initially, glucose was oxidized by GOx in a pH 5.1 buffer producing gluconic acid and hydrogen peroxide. The resulting hydrogen peroxide was subsequently detected by Au/GO-NPs in a pH 4 acetate buffer generating a measurable color change corresponding to the concentration of glucose. The method demonstrated consistent linear detection in the 50–700 µM range indicating its effectiveness for glucose sensing in biomedical diagnostics particularly for diabetic care (Fig. 4b and Table 3).

**Table 3:** Analytical parameters and results for glucose determination using GOx in combination with the Au/GO-NPs-based colorimetric nanozyme sensor

Parameter	Value
Range (µM)	50–700

**Linearity**	
Slope	0.0013
Intercept	0.1536
Correlation coefficient (*r*)	0.9982

### 3.8. Selectivity assessment of the GOx-Au/GO-NPs cascade for glucose detection

Ensuring high selectivity is vital for glucose sensing in biological fluids where multiple interferents may coexist. To examine this, the GOx-Au/GO-NP-based system was tested against several physiologically relevant compounds such as citric acid, sucrose, uric acid, lactose, ascorbic acid, and fructose. Each potential interferent was introduced under optimized assay conditions at typical biological concentrations. As shown in Table 4, none of these compounds produced a notable absorbance response when compared to glucose at equivalent levels. This high specificity is attributed to substrate selectivity *via* the glucose oxidase enzymatic activity and the subsequent rapid hydrogen peroxide conversion catalyzed by Au/GO-NPs. The selective response in the presence of comparable sugars and native compounds validates the platform for analysis in complex biological environments.

**Table 4:** Selectivity study of the Au/GO-NP-based colorimetric glucose sensor evaluating the response toward glucose in the presence of common interfering species

Interferent	Concentration (µM)	Relative response (%)
Glucose	200	91.62
Citric acid	200	2.5
Sucrose	200	4.7
Lactose	200	5.3
Uric acid	200	2.2
Ascorbic acid	200	5.9
Fructose	200	4.1

### 3.9. Glucose quantification in human serum samples

To validate the practical utility of the GOx-Au/GO-NPs assay, it was applied to determine glucose concentrations in commercially available human serum samples. The serum was diluted with a pH 4.0 acetate buffer to align with the linear detection range of the assay. The standard two-step cascade approach, enzymatic glucose oxidation followed by Au/GO-NP-mediated colorimetric detection was employed. Glucose levels measured with the developed sensor were evaluated against a standard diagnostic kit (Spectrum GLUCOSE-Liquizyme-GOD). The sensor reported a concentration of 4.96 ± 0.05 mM compared to 4.77 ± 0.03 mM from the commercial kit. The small discrepancy of 0.19 mM was statistically assessed using a paired *t*-test which produced a *t*-value of 4.94 and a *p*-value of 0.008 indicating strong agreement. These results demonstrate the reliability and precision of the sensor for clinical glucose monitoring.

### 3.10. Performance consistency and durability of Au/GO-NPs biosensor

To assess operational consistency and durability, reproducibility and stability of the biosensor were systematically evaluated. Reproducibility tests were performed by measuring hydrogen peroxide at concentrations of 10, 300, and 600 µM using three individual Au/GO-NPs sensors producing RSDs of 0.14%, 1.05%, and 1.29%, respectively confirming excellent consistency across different batches. Long-term stability assessments over six months demonstrated sustained peroxidase-like activity. These results reflect a steady OPD oxidation in the presence of hydrogen peroxide which underscores the reliable performance and long-term applicability of the biosensor in clinical and point-of-care glucose monitoring scenarios.

## 4 Conclusion

This study successfully synthesized and characterized Au/GO-NPs which exhibited notable peroxidase-mimetic activity. Comprehensive characterization using SEM, TEM, EDX, and FTIR confirmed the effective integration of carbon, oxygen, and gold into a carbon-rich graphene oxide (GO) framework. The catalytic assessment of Au/GO-NPs demonstrated strong catalytic capability in OPD oxidation in the presence of hydrogen peroxide with optimal performance achieved at pH 4, 40 °C, and a 10 minutes incubation. The developed colorimetric platform demonstrated excellent sensitivity and a broad linear detection range for both hydrogen peroxide and glucose with low limits of detection. The cascade detection approach combining glucose oxidase (GOx) with Au/GO-NPs provided accurate and selective glucose quantification even when tested with complex biological matrices such as human serum and was validated through recovery experiments.

In summary, Au/GO-NPs represent a promising, low-cost, and environmentally benign alternative to natural peroxidases. Their stability, biocompatibility, and effective catalytic function suggest strong potential for integration in biosensing systems, medical diagnostic tools, and portable point-of-care technologies.

## Ethical statement

All experimental procedures involving human serum samples were approved by the Ethics Committee of Future University in Egypt and were conducted in accordance with the ethical principles of the World Medical Association Declaration of Helsinki. Human serum samples were purchased under formal authorization from VACSERA (The Holding Company for Biological Products and Vaccines, Giza, Egypt). Informed consent was obtained from all donors by the supplying institution.

## Author contributions

Mohamed A. Abdelgawad, Mohammed Gamal, Jumana A. Sanari, and Ola G. Hussein contributed equally to conceptualization, methodology, software, validation, formal analysis, investigation, resources, data curation, original draft preparation, visualization, supervision, project administration, and manuscript review and editing. Mohamed A. Abdelgawad: funding acquisition. All authors have read and agreed to the published version of the manuscript.

## Conflicts of interest

The authors declare no conflicts of interest.

## Data Availability

All relevant data are included within the article.
